# Synthesis, X-ray Diffraction Study and Antimicrobial Activity of Calcium Sulphate Nanocomposites from Plant Charcoal

**DOI:** 10.3390/ma2020345

**Published:** 2009-04-14

**Authors:** Chira R. Bhattacharjee, Satya B. Paul, Abhijit Nath, Pinak P. N. Choudhury, Sudip Choudhury

**Affiliations:** 1 Department of Chemistry, Assam University, Silchar, Assam-788011, India; E-Mails: dr.sbpaul@gmail.com (S.P.); crbhattacharjee@rediffmail.com (C.B.); 2 Department of Chemistry, S. S. College, Hailakandi, Assam-788151, India; E-Mail: abhijitnath1974@rediffmail.com (A.N.); 3 Department of Zoology, S. S. College, Hailakandi, Assam-788151, India; E-Mail: pinaknath@yahoo.com (P.C.); 4 Department of Chemistry, G. C. College, Silchar, Assam-788005, India

**Keywords:** Charcoal, nanocomposite, XRD, antimicrobial activity

## Abstract

Calcium sulphate nanocomposite materials (CB) have been synthesised from plant charcoal. Crushed charcoal powder was heated to red hot over a Bunsen burner flame and produced a white material which has been isolated. The surface morphology of the material has been studied by Scanning Electron Microscopy (SEM) and the elements were analyzed by Energy Dispersion Spectroscopy (EDS). To explore the structural features of the materials X-Ray Diffraction (XRD) patterns were recorded. The material showed pronounced inhibitory effects against *Streptococcus faecaelis, Bacillus subtilis, Klebsilla pneumoni, E. coli, Proteus vulgaris* and *Pseudomonas aeruginosa*.

## 1. Introduction

Despite the development of a variety of approaches for the synthesis of nanomaterials [1,2], production of such materials in commercial volumes at viable market prices is yet a challenging task. For the preparation of nanomaterials from carbonaceous sources subjecting the starting material to high temperatures has been a common approach. The vapourised material, which is rich in materials in the nano domain, is condensed and collected. Most often, not much attention is paid to the leftover solid residue. In this work, our focus was to study that nonvolatile leftover solid residue from charcoal. Charcoal is made by charring wood in the absence of air [3].

In normal wood ash, the major component is calcium carbonate [4]. Mishra and co-workers reported a very good account of wood ash composition as the function of furnace temperature [5]. They reported the presence of CaCO_3_, K_2_Ca(CO_3_)_2_, K_2_Ca_2_(SO_4_)_3_ at 600 °C and CaO, MgO and Ca_2_SiO at 1,300 °C. Ferrez and co-workers reported the synthesis of nano-hydroxyapatite microsphere (75-106 μm) by the treatment of calcium hydroxide with orthophosphoric acid and their potential as a delivery system for antibiotics [6]. A number of inorganic salts including calcium sulphate, calcium carbonate, copper carbonate etc, when used as additives, have been reported to enhance efficacy of *Beauveria bassiana* and *Metarhizium anisopliae* against the potato tuber moth *Phthorimaea operculella* (Zeller) [7].

We report herein the nano-structured inorganic nanocomposite material, CaSO_4_ obtained from such charcoal-remainings and its antimicrobial properties. CaSO_4_ is well known for its binding and adsorption activity. The presence of Ca^2^^+^ can influence the deposition of sulfate functionalized latex microspheres of natural organic matter [8]. The effect of particle size and natural organic matter on the latex particles migration in saturated porous media was studied by Pelley and Tufenkji [9]. Southam and his coworkers successfully utilized nanostructured calcium silicate for chemisorption of all forms of orthophosphate from aqueous solution [10,11]. Nanostructured calcium silicate consisting of nanometer-sized platelets arranged in particles (0.5 and 20 μm in size) seems to be efficient for treatment of aqueous waste containing phosphate and other ions [11]

## 2. Results and Discussion

The material (coded as CB) was prepared as a white powder residue from the heating of plant charcoal to a red hot state. The white powder was used ‘as obtained’ (see Section 3.1). The morphology of the material was studied using Scanning Electron Microscopy (SEM) and analyzed in regards to its elemental composition by Energy Dispersive Spectroscopy (EDS). The antimicrobial activity of the material was studied by monitoring the zone of inhibition in sterile Muller Hinton agar, cooled to 45 °C.

### 2.1. SEM Studies

**Figure 1:**
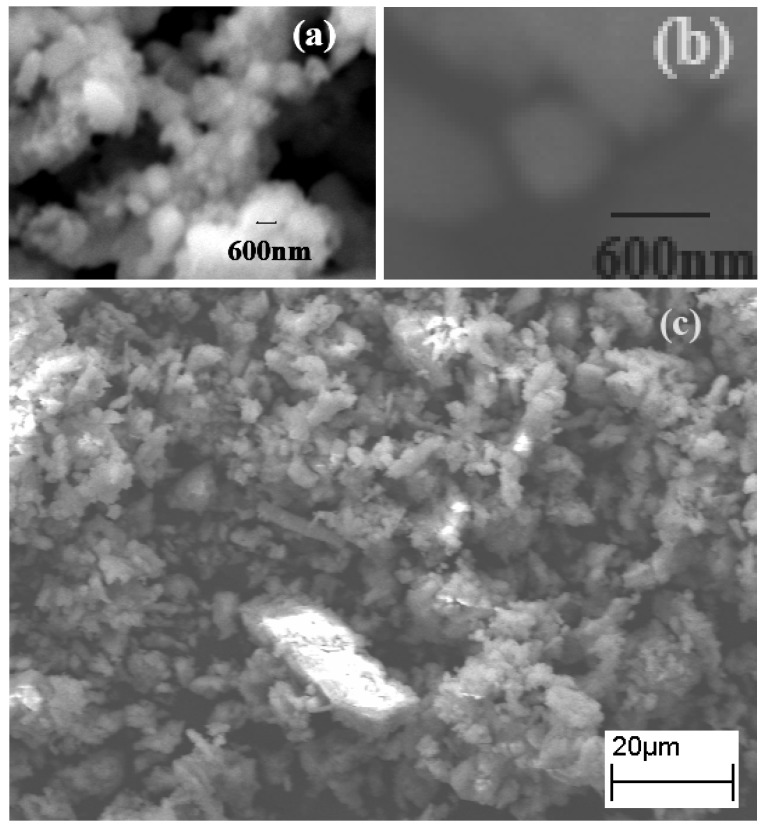
(a) Scanning Electron Microscopy (SEM) images of the synthesized material, CB; (b) single particles; (c) lower resolution image showing aggregates of CB.

The SEM images show the presence of spherical nano-scaled domains forming micro-scale aggregates (Figure 1). The size of the spherical particle has been evaluated to be ~600 nm.

### 2.2. Elemental Analysis

**Figure 2:**
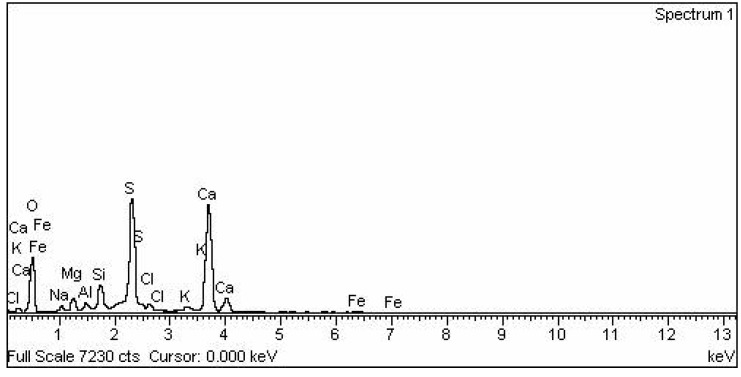
EDS spectrum of the synthesized material, CB.

Energy Dispersion Spectroscopy (EDS) was performed to analyse the elemental composition. The study revealed the presence of oxygen, sulphur and calcium as the major constituents, along with minor contributions from sodium, magnesium, aluminium, silicon, chlorine, potassium and iron. The nitrogen content in wood ash is normally insignificant due to the conversion of most of the wood nitrogen to NH_3_, NO_X_ and N_2_ during the combustion of wood [5]. The percentage composition is given in Table 1 and the EDS spectrum is displayed in the Figure 2.

**Table 1:** Elemental composition of the material CB.

Element	App Conc.	Intensity	Weight%	Weight% Sigma	Atomic%
O	37.55	0.4370	49.94	0.78	68.23
Na	1.55	0.6542	1.38	0.18	1.31
Mg	2.30	0.6318	2.12	0.15	1.90
Al	1.13	0.7350	0.89	0.11	0.72
Si	4.65	0.8396	3.22	0.15	2.50
S	25.86	0.9334	16.10	0.34	10.97
Cl	1.49	0.7180	1.21	0.12	0.75
K	1.27	1.0228	0.72	0.10	0.40
Ca	39.14	0.9582	23.74	0.43	12.94
Fe	0.95	0.8093	0.68	0.15	0.27
Total			100.00		

### 2.3. XRD Studies

**Figure 3:**
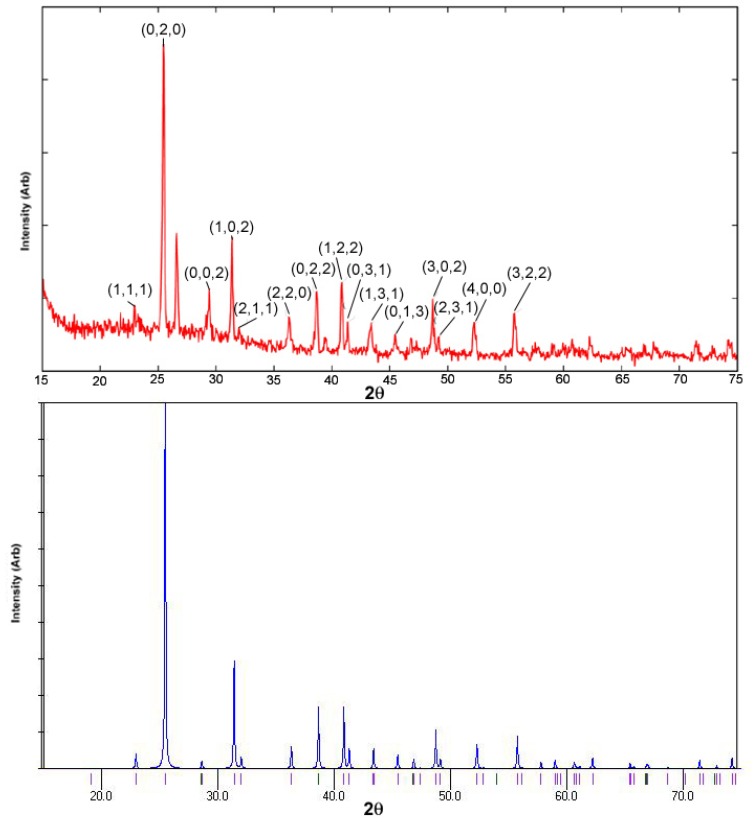
X-Ray diffraction (XRD) pattern of the material, CB. At the top the experimental (red) and the bottom the simulated powder pattern (blue) for CaSO_4_ are plotted.

The X-Ray diffraction (XRD) pattern of the material is presented in Figure 3. The XRD pattern (and the data from EDS study) of the sample indicated the material to be mainly the CaSO_4_ phase. The EDS studies indicated the atomic ratio of calcium and sulphur to be approximately 1:1, along with a high percentage of oxygen. The structure was tried to be resolved from the powder pattern using WinGX suit [12]. The pattern was found to show high similarity with the powder pattern simulated from the single crystal data of CaSO_4_ with space group *A m m a* [13,14] using Mercury 1.4.1 suit [15]. Both the peak position and the intensity ratio exhibited a close match. The experimental (with corresponding Miller Indices) and the simulated patterns are presented in Figure 3 for comparison. The presence of the CaSO_4_ phase also explains the high percentage of oxygen. The rest amount of oxygen seems to arise from other minor metal oxides, which are responsible for the minor extra reflections in the pattern (the unindexed reflections at 27 and at 39.5 [2θ]). No detectable evidence of the presence of CaO was found in the XRD pattern of the sample.

### 2.4. Study of Antimicrobial Activity

The activity of the material against different microorganisms was monitored with the as prepared sample. Disc diffusion method was used to determine the zone of inhibition. The material exhibited a very good inhibitory effect against *Streptococcus faecalis* and *Bacillus subtilis* and lesser activity against *Klebsilla pneumonae*, *E. coli*, *Proteus vulgaris* and *Pseudomonas aeruginosa* relative to standards ciprofloxacin and clotrimazole (Table 2). The solvent chloroform has no antimicrobial activity as such. The antimicrobial property of the material seems to arise due to changes in the microenvironment in the vicinity of organism-particle contact area causing damage to the cell membranes on intimate contact between the cell and particle [16].

**Table 2:** Antimicrobial activity of the material CB.

Sl No.	Microbe	Standard (Zone of inhibition)	Solvent (CHCl_3_)	CB (Zone of inhibition)
1	*Streptococcus faecaelis*	40 mm	Nil	18 mm
2	*Bacillus subtilis*	32 mm	Nil	20 mm
3	*Klebsilla pneumoni*	30 mm	Nil	12 mm
4	*E. coli*	35 mm	Nil	13 mm
5	*Proteus vulgaris*	40 mm	Nil	12 mm
6	*Pseudomonas aeruginosa*	30 mm	Nil	12 mm

Standards used: Ciprofloxacin 10 μg/disc, Clotrimazole 10 μg/disc.

**Figure 4:**
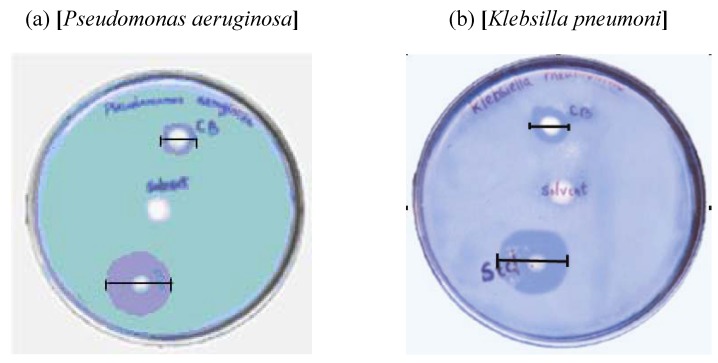
Photographs of two culture plates (the zone of inhibition is marked).

## 3. Experimental Section

### 3.1. Preparation of the materials

A block of plant charcoal (approx. 1.5 g), finely crushed into powder, was heated over a Bunsen burner flame to red hot for two hours. Carbon and other volatile oxidisable materials were removed under these conditions. A white powder residue remained after heat treatment, which was analyzed ‘as obtained’. The morphology of the material was studied using Scanning Electron Microscopy and Energy Dispersive Spectroscopy. The antimicrobial activity of the material was studied by monitoring the zone of inhibition in sterile Muller Hinton agar, cooled to 45 °C

### 3.2. Study of antimicrobial activity

The disc diffusion method was used to determine the inhibition zones. Sterile molten Muller & Hynton agar cooled to 45°C was inoculated with different organisms. The inoculums used were the young cultures and the inoculum size was standardized in such a way that each mL contains 108 cells. Using an aseptic technique the inoculum was uniformly inoculated over the molten agar with sterile cotton swabs. A Watmann No 2 filter paper disc of 6 mm diameter containing 200 µL/disc of sample was placed over the inoculated medium. The plates were allowed to remain at room temperature for two hours. Then the plates were incubated at 37°C for 24 hours. The zone of inhibition was measured using a Zone Reader.

### 3.3. X-Ray Diffraction (XRD) Study

A capillary filled with the sample was used for XRD studies. The powder was finely ground to ensure random orientation of the crystals so that there are detectable signals at all angles and that the background noise is kept to a minimum. The samples were analyzed using a copper target to generate X-rays of 0.154 nm wavelength.

## 4. Conclusions

In conclusion, we have prepared calcium sulphate nanocomposite antimicrobial materials from the nonvolatile fraction of charcoal in an inexpensive way and evaluated their activity against a number of microorganisms. As the materials, *as prepared*, exhibited good microbial inhibitory effect, a more focused effect may be obtained by functionalizing the surface of the material particles. Currently the evaluation of the composite materials as drug carrier is in progress.
